# Optimization of culture medium for *in vitro* germination and storage conditions of *Exochorda racemosa* pollen

**DOI:** 10.3389/fpls.2022.994214

**Published:** 2022-10-11

**Authors:** Wenqing Jia, Yanli Wang, Zhaorong Mi, Zheng Wang, Songlin He, Dezheng Kong

**Affiliations:** ^1^ College of Forestry, Henan Agricultural University, Zhengzhou, China; ^2^ Postdoctoral Research Base, Henan Institute of Science and Technology, Xinxiang, China

**Keywords:** *Exochorda racemosa*, ultra-morphology, pollen germination, storage, pH, temperature

## Abstract

Pollen morphology, pollen vigor, and long-term pollen storage are critical for plant cross-breeding and genetic improvement of *Exochorda racemosa*. We developed a protocol for viability determination and storage of *E. racemosa* pollen for breeding new varieties. The medium components for *E. racemosa* pollen germination was optimized by using an Orthogonal Assay Test Strategy (OATS). The germination rates of *E. racemosa* pollen were investigated after storing at different temperatures and different storage periods. The size of *E. racemosa* pollen was medium with three germination ditches, and the sculptural type of pollen was striate. Red ink and 2,3,5-triphenyl tetrazolium chloride (TTC) can effectively distinguish viable pollen from the unviable pollen of *E. racemosa*. The most suitable medium (CK2) for *E. racemosa* was composed of 150 g· L^–1^ sucrose, 100 mg·L^–1^ boric acid, 150 mg· L^–1^ Ca(NO_3_)_2_ and 50 mg· L^–1^ GA_3_. Low-temperature stress produced the greater inhibition of pollen tube growth compared with high-temperature conditions. The CK2 medium at pH 6.5 resulted in the highest pollen germination rate and most extended pollen tube length. The optimal temperature for storage of dried pollen was –80°C (*P* < 0.01), and the germination rate was 53.60% after storage for 390 days. Thawing in a 35°C water bath produced the best viability of *E. racemosa* pollen after storage at –20°C and –80°C. The short-term storage of *E. racemosa* fresh pollen at 4°C was better than that at –20°C and –80°C (*P* < 0.01). It is possible to evaluate pollen quality and store pollen grains for *E. racemosa* by the parameters defined in this study.

## Introduction

*Exochorda racemosa* (Lindl.) Rehder (Rosaceae) is a 2 to 5 m tall tree species native to China. *E. racemosa, E. serratifolia* and *E. giraldii *are also named pearlbush because their inflorescence resembles a string of pearls (Schneider, 2012; Kim et al., 2014; Song et al., 2016; Zhang et al., 2018). *E. racemosa* has white blooms with fan-shaped petals in spring, and its fruits are golden yellow, five-sided capsules (Zhang et al., 2011; Palancean et al., 2015; Zhang et al., 2018). The flowers and tender leaves of *E. racemosa* are used as a high-quality food (Zhang et al., 2018). In addition to having high ornamental, food and medicinal value, *E. racemosa* is also resistant to heat, cold and drought.

Hybridization is an important means of plant speciation (Soltis and Soltis, 2009; Goulet et al., 2017), and it also contributes to increasing intraspecific genetic diversity and the transfer of genetic adaptations (Abdullah Yousuf Akhond et al., 2000; Soltis and Soltis, 2009). Each of the 6-20 key species in the genus *Rosa* contributes a specific trait to modern rose cultivars through hybridization. For example, *Rosa gallica* and other robust polyploid species lend the trait of cold hardiness, and *Rosa chinensis* brings recurrent blooming (Bendahmane et al., 2013). *E. racemosa* is a relatively common garden plant with high ornamental value, but is susceptible to powdery mildew (Zhang et al., 2018). Therefore, it is urgent to genetically improve the powdery mildew resistance of *E. racemosa* through hybridization. However, studies on pollen viability, fertilization ability and storage are very important for hybrid breeding programs, as pollen quality is a key factor in seed set (Wood, 2017; Kaur and Singhal, 2019). Pollen viability, especially the staining rate obtained by the pollen staining method, is not always correlated to the germination rate because pollen may retain metabolic capacity but lose germination ability (Wang et al., 2021). Therefore, the best method to evaluate pollen viability may be a combination of staining and germination test. A germination test is an effective and convenient method to study the applied aspects of pollen biology (Atlagić et al., 2012; Jia et al., 2015; Alexander, 2019). The pollen germination is highly sensitive to not only media components including calcium (Wood, 2017; Sorkheh et al., 2018), boron ions (Shekari et al., 2016; Gokbayrak and Engin, 2017) and sugar (Jia et al., 2020; Jia et al., 2021), but also environmental factors such as temperature (Memmott et al., 2007; Milatović et al., 2015; dos Santos Ferreira et al., 2021) and humidity (Iovane et al., 2021). The pH of the *in vitro* germination medium can also affect pollen germination and pollen tube growth in many plants (Therios et al., 1985; Burke et al., 2004; Mbogning et al., 2007).

Efficient pollen storage is important for artificial pollination, hybrid breeding and dealing with asynchronous flowering (Mesnoua et al., 2018a; Jia et al., 2021). The natural longevity of fresh pollen is typically only 5–15 days, which does not meet the needs of most hybridization procedures. Improving pollen storage methods can help prolong the lifespan of pollen and solve the problem of hybridizing plants with asynchronous flowering (Jia et al., 2020).

Previous research on *E. racemosa* has mainly focused on medicinal value, edible value and tissue culture (Yang and Kamp-Glass, 2000; Lee et al., 2006; Zhang et al., 2011; Zhang et al., 2018). Pollen quality is an important component of pollination biology and is significant in the selection of pollinating species and the identification of pollen pistils and fertilization (Liu et al., 2020). There is little information available on *E. racemosa* pollen quality and its determination. Although reference can be made to the pollen viability of other plant species, appropriate methods for measuring pollen viability for *E. racemosa* must be developed according to its specific pollen characteristics.

Protocols for determining *E. racemosa* pollen viability need to be optimized to perform controlled pollination and evaluate intra- and inter-cultivar incompatibility. Protocols are also needed to arrange the pollinating plants (varieties) for the main cultivars in orchards, as well as for clonal selection and genetic breeding trials. Our goal was to determine the optimal media for *E. racemosa* pollen germination and optimal conditions for pollen storage of *E. racemosa*. The objectives of this study were therefore to: (1) determine the optimal culture medium condition for *in vitro* pollen germination; (2) analyze the relationship between pollen viability estimates obtained through staining and estimates obtained from germination; and (3) determine the best *in vitro* conditions for pollen storage. The results of this study provide technical support for artificial pollination breeding and pollination tree configurations.

## Materials and methods

### Pollen collection

The flower buds of 8–10 years old *E. racemosa* at the pearl stage were collected from the Grand Canyon of Taihang Mountains in Northern China, and brought back to the laboratory. The anthers were isolated from the flower buds with tweezers and put in Petri dishes, which were later placed in a climatic chamber at 25°C to release pollen grains. After 36h, the fresh pollen grains released from anthers were filtered to eliminate waste using a metal mesh with 50 μm^2^ openings. Subsequently, the fresh pollen grains were collected and divided into two parts: one part was used to determine the pollen germination rate, tube length and morphology, and another for preservation.

### SEM observation on pollen morphology

The pollen grains were dehydrated in an air dry oven at 45°C for six hours, and then spread on the sample stage with black double-sided cellophane tape and coated with gold by a sputter coater. The digital micrographs were taken by a SEM (FEI Quanta 200 scanning electron microscope) to analyze the shape and sculpture of pollen grains. The photos of pollen grains were taken at 500–3500 times magnification, and the polar and equatorial diameters of 30 randomly selected pollen grains were measured. The description of pollen morphology was mainly based on the glossaries described by Punt et al. (2007) and Halbritter et al. (2018).

### Optimization of culture medium for *in vitro* pollen germination

An orthogonal experimental design (L_9_[3]^4^) was used to optimize the germination medium for *E. racemosa* pollen compared with a pure water culture medium (**Table 1**, CK1). The number ‘4’ indicates four factors ([1] sucrose, [2] H_3_BO_3_, [3] GA_3_ and [4] Ca(NO_3_)_2_), ‘3’ means three levels ([1] sucrose (120, 150, and 180 g· L^−1^), [2] H_3_BO_3_ (80, 100, and 120 mg· L^−1^), [3] GA_3_ (50, 75, and 100 mg· L^−1^) and [4] Ca(NO_3_)_2_ (50, 100, and 150 mg· L^−1^)), and ‘9’ means nine treatments (**Table 1**, M_1_–M_9_). All components of the medium for each treatment were dissolved in distilled water to prepare a liquid medium. Fresh pollen grains (150-200) were placed in a drop of liquid medium (50 μL) on a coverslip which was then inverted over the concave of a concave glass slide, and the slide was placed in Petri dishes covered with absorbent cotton soaked with water (hanging drop technique) (Addicott, 1943). The Petri dishes were incubated at 24°C in a humidity chamber under dark conditions. The germinated pollen grains were counted under a fluorescence microscope (BX53, Olympus, Japan) (10× magnification) after 5h of incubation. The pollen germination rate was determined by counting the number of germinating pollen grains of three slides (two concaves per slide). A pollen grain was considered germinated if the length of the pollen tube was longer than the diameter of the pollen grain (Jia et al., 2020). The pollen germination rate was calculated based on the formula: germination rate = (germinated pollen/total number of pollen grains) × 100.

**Table 1:** Germination media of *E. racemosa* pollen grains in an orthogonal design assay.

Treatment	Sucrose (g·L^−1^)	Boric acid (mg·L^−1^)	Ca(NO_3_)_2_(mg·L^−1^)	GA_3_(mg·L^−1^)	Germination rate (%)
M_1_	120	80	50	50	61.54 ± 1.65 C
M_2_	120	100	100	75	65.18 ± 1.05 B
M_3_	120	120	150	100	54.05 ± 1.50 DE
M_4_	150	80	100	100	67.05 ± 1.02 B
M_5_	150	100	150	50	80.18 ± 2.52 A
M_6_	150	120	50	75	65.20 ± 1.01 B
M_7_	180	80	150	75	53.54 ± 1.10 E
M_8_	180	100	50	100	56.66 ± 1.08 D
M_9_	180	120	100	50	61.68 ± 2.14 C
CK1	0	0	0	0	15.68 ± 1.68 F
K1	60.50 B	60.71 B	61.13 B	67.15 A	
K2	70.81 A	67.58 A	64.88 A	61.55 B	
K3	57.29 C	60.31 B	62.59 B	59.25 B	
*R*	12.17	6.55	3.38	7.70	

Data shown are the mean ± SD. Different capital letters indicate significant differences at the 0.01 level. K1, K2, and K3 indicate the average pollen germination rate of E. racemosa at three levels of each factor; R represents the extreme difference at the level of the same factor.

### Pollen staining

Pollen viability was determined by seven staining methods, including red ink (Brilliant crocein + Acid red 87) (Chen et al., 2010), carmine acetate (Mazzeo et al., 2014), TTC (2,3,5-triphenyl tetrazolium chloride) (Luo et al., 2020), MTT (2,5-diphenyl monotetrazolium bromide) (Rodriguez-Riano and Dafni, 2000), I_2_-KI (iodine + potassium iodide) (Du et al., 2019), Alexander’s stain (Alexander, 1969) and fluorescein diacetate (Dafni, 1992) (**Table 2**). Seven staining solutions were prepared according to **Table 2**. Two drops of staining solution were put on a glass slide and pollen samples were sprinkled on the drop with a slim brush. Then, the drop was carefully covered by a coverslip without trapping air after staining for 5**–**20 min (**Table 2**) under dark conditions. Viable pollen grains were counted under a fluorescence microscope (BX53, Olympus, Japan) (10× magnification). The deeply stained (**Table 2**) and normal pollen grains for each staining method were considered to be viable, whereas shriveled, lightly stained or colorless pollen grains were counted as non-viable. For each staining method, the pollen viability rate was determined using three glass slides (at least 200 grains per slide).

**Table 2:** Method to assess pollen viability.

Staining method	Preparation of staining solution	Staining time (min)	Viable color or response
Red ink	1% red ink (Hero)	5	Red
Carmine acetate	1% carmine acetate: 1.0 g carmine acetate (Sigma) dissolved in 100 mL distilled water	5	Red
TTC	0.5% TTC: 0.5 g TTC (Sigma) dissolved in 100 mL 95% alcohol	20	Red
MTT	0.3% MTT: 0.3 g MTT (Sigma) dissolved in 100 mL PBS	20	Violet-purple
I_2_-KI	0.5% I_2_-KI (Macklin): 80 g potassium iodide KI and 10 g iodine I dissolved in 100 mL distilled water	20	Blue
Alexander’s stain	10 mL 95% ethanol, 1 mL of 1% solution malachite green in 95% ethanol, 25 mL glycerol, 5 mL of 1% acid Fuchsin in water, 0.5 mL of 1% solution Orange G in water, 4 mL glacial acetic acid, and distilled water to a total of 100 mL (Macklin)	5	Dark purple
Fluorescein diacetate (FDA)	2 ml 20% saccharose in water with several drops of stock solution of FDA (2 mg FDA/1 ml acetone) (Macklin)	5	Green

### Optimization of pH and temperature for *in vitro* pollen germination and pollen tube growth

The components of the control liquid medium (CK2) were 150 g· L^−1^ sucrose, 100 mg· L^−1^ boric acid, 150 mg· L^−1^ Ca(NO_3_)_2_ and 50 mg· L^−1^ GA_3_. We adjusted only the pH of CK2 (4.5, 5.0, 5.5, 6.0, 6.5, 7.0, 7.5, and 8.0) to investigate the effect of pH on *in vitro* fresh pollen germination and pollen tube elongation. Temperature experiments were carried out in the same way with temperature gradients of 10°C, 20°C, 25°C, 30°C and 35°C. After 24 h of incubation under dark conditions, the pollen germination rate was calculated as stated before. The pollen tube length was measured using a fluorescence microscope (BX53, Olympus, Japan) with the help of Olympus cellSens standard software (Olympus, Japan). Each treatment for pH or temperature experiment contained three replicates. The germination rate and pollen tube length were based on at least 100 pollen grains per replicate.

### Long-term pollen storage

The fresh pollen grains were dehydrated in a drying box at 30°C for 12 h. Pollen grains with an 8–9% water content were categorized as dried pollen. The dried pollen grains and fresh pollen grains (water content of 16–19%) were divided into four parts, and these were stored at 25°C (room temperature), 4°C, −20°C and −80°C. The water content of 0.2 g of pollen grains was determined at 105°C by drying to a constant weight, and the percentage water content of the pollen was calculated (Barnabas and Rajki, 1976). Pollen samples were immersed in liquid nitrogen before storage at −20°C and −80°C. Thirty centrifuge tubes with 0.2 g of pollen grains were placed at each temperature. One centrifuge tube was removed from each storage temperature for pollen germination assays after 5, 10, 30, 50, 70, 90, 120, 180, 270 and 390 days of storage. Stored pollen grains at −20 and −80°C were thawed in a 35°C water bath for 4 min.

All of the stored pollen grains were cultured in a liquid medium consisting of sucrose (150 g·L^−1^), boric acid (100 mg·L^−1^), Ca(NO_3_)_2_ (150 mg·L^−1^) and GA_3_ (50 mg·L^−1^) by the hanging drop technique in an incubator. The conditions were 25 ± 1°C and 50% relative humidity (RH). After 5 h, the pollen germination rate was assessed using a fluorescence microscope (BX53, Olympus, Japan). The design of the germination experiment was completely randomized with three replications (three slides) per storage temperature for each storage time. The germination rates were measured by counting 200 pollen grains for each replication.

### Thawing treatments

Dried pollen grains stored at −20°C and −80°C for 120 days were used as experimental materials to perform four thawing tests, which were 35°C water bath for rapid thawing (4 min), 4°C in a refrigerator for slow thawing (30 min), running tap water (18°C) (30 min), and 25°C for room temperature thawing (30 min), respectively. The pollen germination percentage and tube length were determined after thawing. For each thawing treatment, the experiment was repeated at least three times with three biological replications.

### Statistical analysis

Data concerning pollen viability, germination and pollen tube length are average values of at least three replicates. All data were statistically analyzed by using SPSS 19.0. the data from experiments were expressed as mean values ± standard deviation. Orthogonal analysis of variance was carried out to determine the significance (*P* < 0.01) of the main effects (sucrose, H_3_BO_3_, GA_3_ and Ca(NO_3_)_2_) on pollen germination, means grouping was with Duncan’s test (*p* < 0.01). Data of different temperature, pH and storage time was subjected to one-way analysis of variance (ANOVA). Differences between samples were determined by Fisher’s least significant difference (LSD) test (*P* < 0.01). Moreover, the correlation between pollen viability by staining method and germination rates was statistically evaluated by Pearson’s correlation coefficient (r) at a *P* values of 0.01. All of the figures were drawn using SigmaPlot 14.0 software.

## Results

### Pollen morphological characteristics

**Figure 1:**
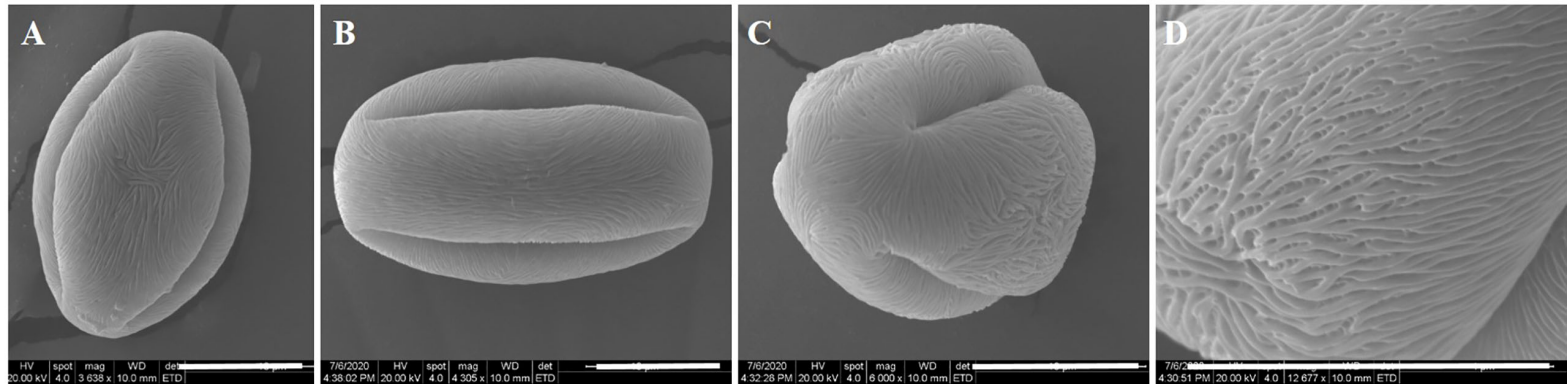
Ultra-morphology of pollen grains of *E. racemosa* under the SEM. **(A, B)** Equatorial view; **(C)** Polar view; **(D)** Exine sculptures. Bar = 10 μm.

The shape and sculpture of pollen are useful characteristics for the identification of species and the determination of interspecific relationships in Exochorda spp. The average length of pollen grains of E. racemosa was 32.50 ± 3.67 μm, and the average width of the equatorial diameter was 15.60 ± 1.85 μm. The ratio of the length of the polar axis (P) to the equatorial diameter (E) in E. racemosa pollen was ≈2, so the shape of E. racemosa pollen was spheroidal, and the pollen size was medium. The pollen grains had three zonicolpate apertures, each with a width of approximately 3 μm from polar view, which dehisced from one pole to the other along the longitudinal axis (**Figures 1A–C**). The pollen had striate sculpturing (**Figure 1D**). Malformed and underdeveloped pollen accounted for 16.56% of the total pollen grains.

### Optimization of pollen germination medium for *E. racemosa*

**Figure 2:**
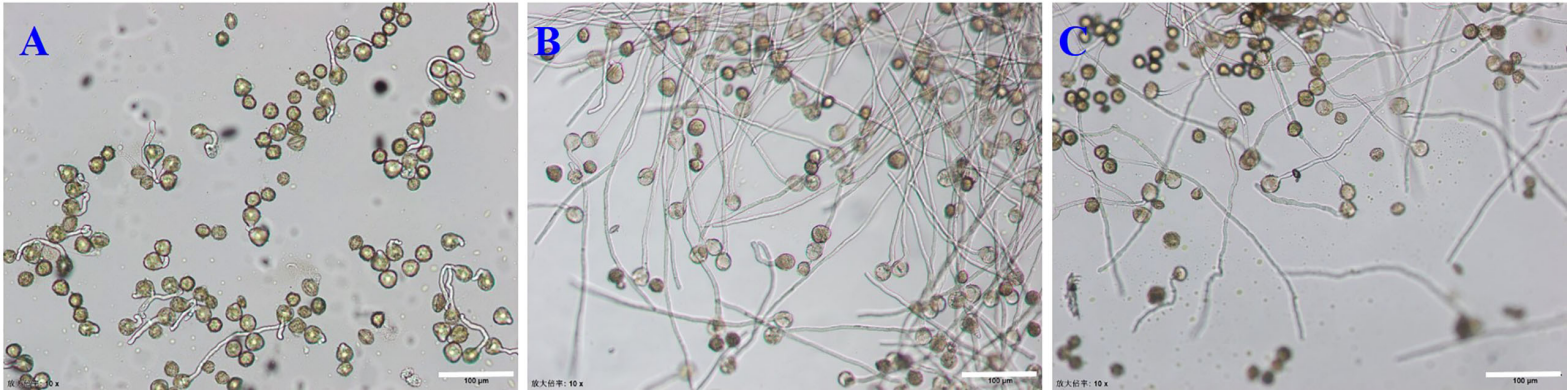
Germination rate of *E. racemosa* pollen in different culture media. **(A)** The CK1 medium; **(B)** The M_5_ medium; **(C)** The M_7_ medium. Bar = 150 μm.

Sucrose concentration had a significant effect on pollen germination *in vitro* (*P* < 0.01), and there was an interaction between factors. Sucrose had the most significant effect (R = 12.17) on pollen germination, with an average range of 57.29–78.81%. The medium with 150 g· L^−1^sucrose promoted higher germination compared to the medium containing 120 g·L^−1^ sucrose, 180 g·L^−1^sucrose or no sucrose (**Figures 2A–C**). This indicated that sucrose was important in pollen germination. A germination rate of 80.18% occurred in M_5_ medium, while in the pure water medium (CK1) **(** **Table 1** **)**, the germination rate was only 15.68% (**Figures 2A, B** **)**. Based on the ANOVA, sucrose was the most important factor affecting pollen germination, followed by GA_3_, boric acid and Ca (NO_3_)_2_ (**Tables 1**, **3**). The second level of sucrose, Ca(NO_3_)_2_ and boric acid had the highest average value of the three replications (xx = 70.81%, 67.58% and 64.88%, respectively) compared to their first and third levels (**Table 1**). GA_3_ had the highest average value (xx = 67.15%) at the first level compared to the second and third levels (**Table 1**).

**Table 3:** ANOVA results for the effects of different media on pollen germination of *E. racemosa*.

Source	Variance of square	df	Average of square	F value (*F*)	*P* value
Sucrose	545.29	2	272.64	74.49**	0.0001
Boric acid	284.43	2	142.21	38.86**	0.0001
Ca(NO_3_)_2_	32.13	2	16.06	4.39*	0.0467
GA_3_	344.55	2	172.27	47.07**	0.0001
Standard error	32.94	9	3.66		
SUM	1206.38	8	150.80	41.20	0.0001

"*" indicates significant differences (*P* < 0.05). "**" indicates very significant differences (*P* < 0.01).

### Optimization of pollen viability estimates for *E. racemosa*

**Figure 3:**
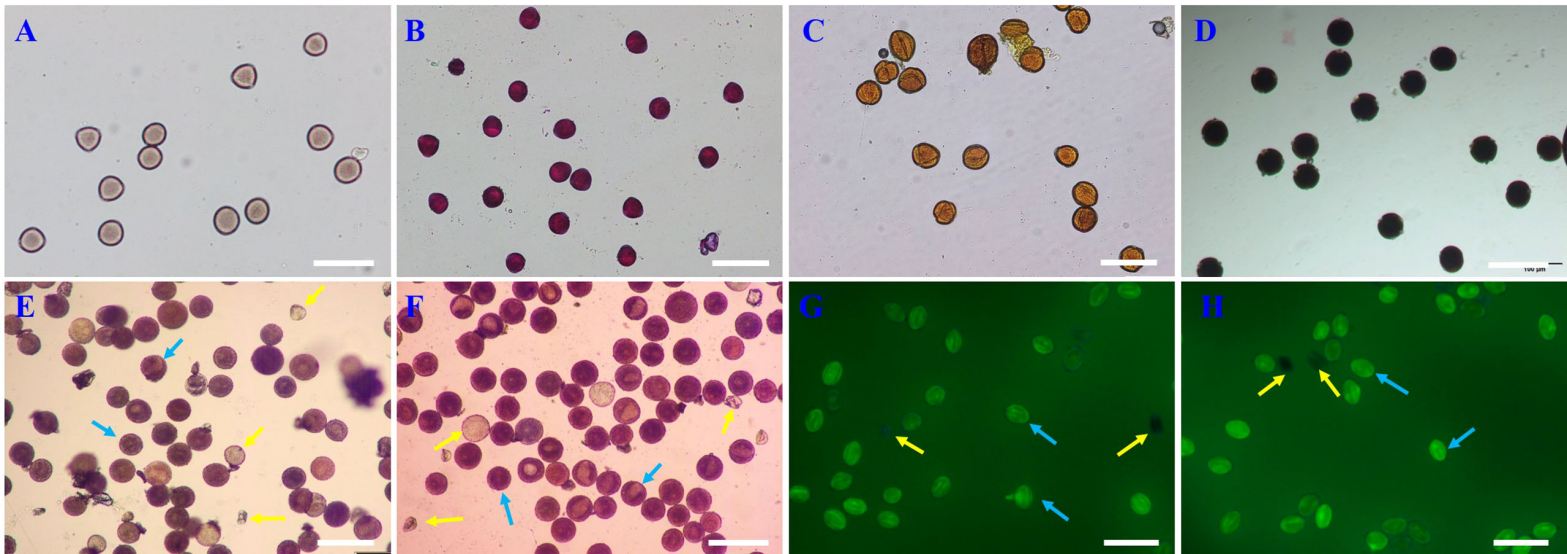
Comparison of the seven staining methods for *E racemosa* pollen. **(A)** Carmine acetate; **(B)** Alexander; **(C)** I-KI_2_; **(D)** MTT; **(E)** TTC; **(F)** Red ink; **(G, H)** fluorescein diacetate. The yellow arrow indicates non-viable pollen; the blue arrow indicates viable pollen. Bar = 100 μm.

Using existing staining methods, i.e., staining with magenta acetate, Alexander’s stain, I_2_-KI and MTT, did not distinguish between viable and non-viable pollen (**Figures 3A–D**). TTC, red ink and fluorescein diacetate can effectively distinguish viable pollen from the unviable pollen for E. racemosa (**Figures 3E–H**). For TTC staining, the viable pollen was dyed red (**Figure 3E**) with 79.6% pollen viability, which was similar to the pollen germination (80.18%). For the red ink stain method, red coloring was indicative of viable pollen grains with 82.30% pollen viability, which was similar to the pollen germination (80.18%), whereas the non-viable pollen grains were shriveled or lightly stained (**Figure 3F**). Fluorescein diacetate gave the highest pollen viability rates(86.25%) (**Figures 3G, H**), which was statistically different from actual pollen germination (80.18%). The correlations between pollen viability and *in vitro* germination tests were determined. A correlation matrix indicated that the germination rate of pollen grains was positively correlated with pollen viability by the TTC, red ink and fluorescein diacetate staining tests (**Table 4**). There were no significant differences (*P* < 0.01) in viability measured by TTC and red ink staining and *in vitro* germination test.

**Table 4:** Correlation matrix between the *in vitro* pollen germination rate (%) and the pollen viability.

	Germination rate	TTC	Red ink	Fluorescein diacetate
Germination rate	1.000			
TTC	0.862**	1.000		
Red ink	0.538**	0.759**	1.000	
Fluorescein diacetate	0.623**	0.815**	0.884**	1.000

“**” indicates that the correlation appeared a very significant level.

### Effects of temperature and pH on pollen germination and pollen tube growth

**Figure 4:**
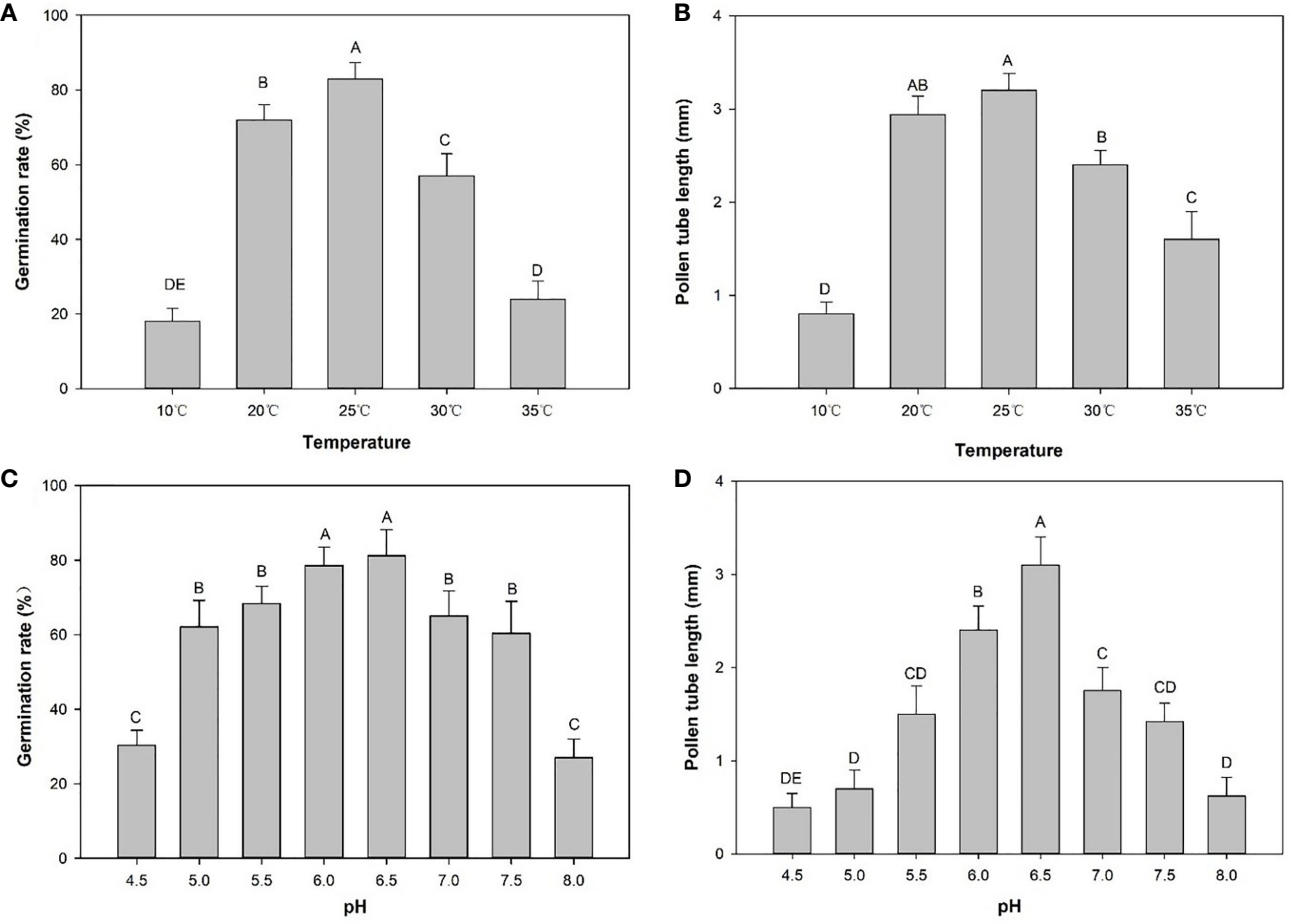
Germination rates (%) and pollen tube length (mm) in CK2 medium at different temperatures or pH values. **(A)** Germination rates at different temperatures. **(B)** Pollen tube lengths at different temperatures. **(C)** Germination rates at different pH values. **(D)** Pollen tube lengths at different temperatures. Distinct letters point out the statistically significant differences (*P* < 0.01) and error bars indicate the standard deviations.

The pollen germination rate and tube lengths were measured to determine the effects of low and high temperatures. Germination rates and tube length of E. racemosa pollen in hanging drop tests exhibited significant differences at different temperatures. The germination rate at 24 h was highest at 82.90% at 25°C, followed by 20°C, 30°C, 10°C and 35°C. Compared to incubation at 25°C, the pollen germination rate at 35°C decreased to 22.60%, while the pollen germination rate only decreased to 72.20% at 20°C. Pollen tube length after 24 h of incubation was highest at 3.2 mm at 25°C (**Figures 4A, B** **)**. However, low-temperature stressed pollen grains exhibited degradation in tube growth. Under low-temperature stress, the tube length was reduced to 2.9 mm at 20°C and 0.8 mm at 10°C. Pollen tube length decreased to 2.6 mm at 30°C and 1.5 mm at 35°C under high-temperature stress. These results demonstrated that low-temperature stress significantly inhibited pollen tube growth compared to high-temperature conditions.

The pollen germination rate initially increased and then decreased as the pH level of the medium increased (**Figure 4C**). There were significant differences in pollen germination rates among the different pH levels. The pH 8.0 treatment produced the lowest (27.00%) pollen germination rate (**Figure 4C**). The pH 6.5 treatment had the highest (81.20%) pollen germination rate. This rate was not significantly different from the germination rate (78.90%) in the pH 6.0 treatment. The longest pollen tube length occurred at pH 6.5 and the shortest pollen tube length was recorded at pH 4.5 (**Figure 4D**).

### Effects of storage temperatures and storage times on pollen germination

The effects of different storage temperatures and two different water contents on pollen germination are presented in **Table 5**. Pollen germination was significantly different following different storage temperatures. For dry pollen, the germination rate of pollen stored at 25°C decreased rapidly with increasing storage time, and pollen lost its viability after 180 days of storage. The pollen germination rate was reduced to 20.5% after storage at 4°C for 390 days. Under storage at −20°C and −80°C, the germination rate of pollen grains slowly decreased. After 390 days, pollen stored at −80°C maintained a germination rate of 53.6%, which was significantly higher than the rate of 40.3% when stored at −20°C. In this study, −80°C was the optimum temperature for storage of dried pollen. Our findings indicated that the longevity of dried pollen at −80°C was longer than 365 days. Similar to dried pollen, the germination rate for fresh pollens also decreased with increased storage time, but the germination rate decreased more rapidly at the four temperatures (**Table 5**). Fresh pollen stored at 25°C lost its viability after 30 days of storage, while pollen grains almost lost most of their viability when stored at 4°C for 70 days (12.0%), at −20°C for 90 days (10.0%), and at −80°C for 120 days (8.9%). The germination rate of dried pollen was significantly higher than that of fresh pollen at different storage times at four storage temperatures. Therefore, it is necessary to reduce the water content of pollen before storage. Compared to 25°C, 4°C and −20°C, −80°C is the optimal storage temperature for dried pollen grains of E. racemosa.

**Table 5:** Effects of storage temperature and storage times on *in vitro* pollen germination.

Storage temperature (°C)	Storage time (Days)										
	5	10	30	50		70	90	120	180	270	390
	**Dried pollen**										
25	61.0B	54.2D	37.2C		18.5D	10.2D	5.3D	2.6D	0.0D	0.0D	0.0D
4	79.2A	73.0C	68.0B		64.0C	60.0C	45.0C	40.0C	36.2C	27.5C	20.5C
−20	77.4A	77.1B	76.6A		72.5B	68.3B	65.6B	60.8B	53.6B	43.2B	40.3B
−80	78.5A	81.5A	78.0A		79.5A	78.0A	75.2A	74.7A	72.5A	60.5A	53.6A
	**Fresh pollen**										
25	36.0D	18.0D	0.0C		0.0C	0.0C	0.0C	0.0	0.0	0.0	0.0
4	66.0A	62.0A	50.0A		26.0B	12.0C	0.0C	0.0	0.0	0.0	0.0
−20	55.0C	46.1C	35.5B		20.8C	20.5B	10.0B	0.0	0.0	0.0	0.0
−80	59.2B	54.3B	47.6A		34.6A	27.8A	20.5A	8.9	0.0	0.0	0.0

Different capital letters indicate very significant difference (*P* < 0.01).

### Effects of thawing method on pollen germination

**Figure 5:**
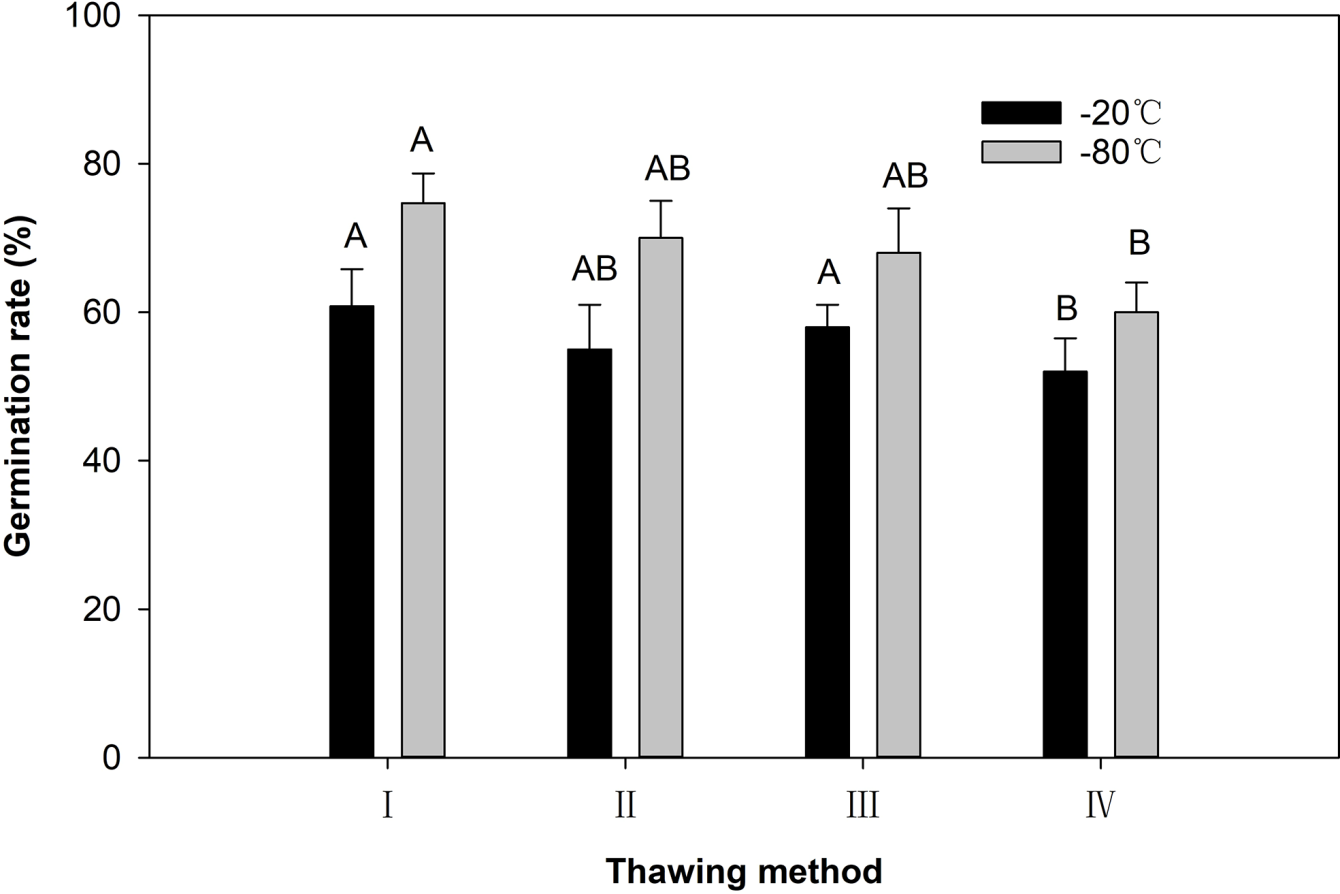
The pollen germination rate after different thawing ways. I: Thawing in a water bath at 35°C for 4 min; II: Thawing in a refrigerator at 4°C for 30 min; III: Thawing with running tap water at 18°C for 30 min; IV: Thawing at room temperature (25°C) for 30 min. Different capital letters indicate very significant difference (*P* < 0.01).

For the dried pollen grains at −80°C after 120 days of storage, the germination rate of pollen after thawing at 35°C was 75.5%. This was significantly higher than that after thawing at 25°C (**Figure 5**). There were no significant differences between the other thawing methods. For dried pollen grains at −20°C after 120 days of storage, the highest pollen germination rate (60.8%) was observed after thawing at 35°C, followed by thawing at 18°C (57.5%), 4°C (55.0%) and 25°C (52.0%) (**Figure 5**). After 120 days of storage at −80°C and −20°C, the pollen germination rate was the highest after thawing at 35°C, and the lowest after thawing at 25°C (**Figure 5**).

## Discussion

### Pollen morphology

The pollen morphological characteristics are typically stable, showing the common characteristics at the family, genera or specie level (Stafford and Knapp, 2006; Jia et al., 2015; Goncalves-Esteves et al., 2021). Walker (1974) reported that the sculptural types of primitive type pollen are simple, and the pollen sculptural types of advanced species are complex. Plant species with striate pollen are generally more advanced (Walker, 1974). Zhou et al. (1999) found that foveolate pollen is the most primitive, and species with striate pollen are more advanced among the nine species of *Spiraea*. We found that the pollen size of *E. racemosa* was medium and the pollen sculptural type of *E. racemosa* was striate which was similar to *Spiraea purpurea* (Lu et al., 2005). Therefore, we speculate that *E. racemosa* may be a more evolved species within the Spiraeoideae subfamily. However, the palynological data of the subfamily Spiraeoideae are poorly understood, and further research is required to clarify the taxonomic status and phylogenetical relationships of *E. racemosa*.

### Pollen germination and pollen staining tests

It is significant to determine pollen germination requirements for cross-pollination (Kremer and Jemrić, 2006; Abdelgadir et al., 2012) because different plants have different requirements for pollen germination (Dane et al., 2004). Sucrose, H_3_BO_3_, Ca^2+^ and GA_3_ are the most commonly used additions for *in vitro* pollen germination assays (Gokbayrak and Engin, 2017; Flores-Rentería et al., 2018). Among them, sucrose not only provides the necessary energy for pollen germination and pollen tube growth, but also maintains the osmotic pressure of the external environment and ensures pollen germination (Hirose et al., 2014). H_3_BO_3_ increases sugar uptake, translocation and metabolism by pollen cells, inducing Ca^2+^ entry from the outside environment and establishing the Ca^2+^ gradient required for the pollen tube tip growth gradient. Ca^2+^ helps regulate the polar growth and growth direction of pollen tubes (Ren et al., 2022). GA_3_ functions in pollen germination and pollen tube growth. Low concentrations of GA_3_ can significantly increase the pollen germination rate in apricot (Gokbayrak and Engin, 2017; Flores-Rentería et al., 2018). Our results showed that the pollen grains still germinated even in the presence of water only, but the germination rate was relatively low (15.68%). A possible explanation is that an ergastic substance stored in pollen provides nutrients for the pollen tube growth. By contrast, the germination rate of *E. racemosa* pollen increased significantly with the addition of sucrose, H_3_BO_3_, Ca^2+^ and GA_3_. These data indicated that the nutrients stored in most *E. racemosa* pollen grains are insufficient to support pollen germination, and the germination of most pollen grains requires exogenous nutrients. The appropriate medium for pollen germination differs widely among plant species (Li et al., 2009; Yu et al., 2010; Luo et al., 2020; dos Santos Ferreira et al., 2021). The orthogonal experiments showed that sucrose and H_3_BO_3_ were the main factors affecting pollen germination, which was consistent with studies on pollen germination of *Paeonia ludlowii* (Jia et al., 2021), *Ketele*eria *fortune* (Mesnoua et al., 2018a) and *Paeonia qiui* (Jia et al., 2020).

Staining is a common method used to estimate pollen viability due to its simplicity and speed. However, staining methods are not suitable for evaluating the pollen viability of all plant species (Impe et al., 2020; Luo et al., 2020). Of the seven staining methods tested in this study, only red ink, TTC and fluorescein diacetate could distinguish viable pollen from unviable pollen, and these showed a high correlation with the fresh pollen germination *in vitro*. The pollen viability indicated by red ink and fluorescein diacetate staining was somewhat higher than the actual pollen germination. These data were consistent with the results of Luo et al. (2020) in *Castanea mollissima* pollen grains and Pearson and Harney (1984) in *Rosa* pollen grains. There were no significant differences between the pollen viability estimated from the TTC test and the pollen germination. These results are consistent with those of Abdelgadir et al. (2012), who noted that TTC is an effective method for determining pollen viability in *Jatropha curcas.*

Plant reproductive development is susceptible to extreme temperatures (Mesnoua et al., 2018a; Sorkheh et al., 2018), and high or low temperatures during the reproductive period can lead to lower pollen germination. This can affect fruit and seed setting (Vasilakakis and Porlingis, 1985; Sukhvibul et al., 2000; Pirlak, 2002; Acar and Kakani, 2010). The optimal temperature for pollen germination of most plants ranges from 20°C to 28°C (Sorkheh et al., 2018; Beltrán et al., 2019; Iovane et al., 2021), and only a few species require higher (Mbogning et al., 2007; Cao et al., 2016; Fan et al., 2018) or lower (Li et al., 2005) temperatures. Excessively high or low incubation temperature can reduce pollen germination. Our study simulates the extreme temperature in spring in the Taihang Mountains, and the main purpose was to study the effect of high or low temperature on the pollen germination rate of *E. racemosa*. The results showed that the germination rate of pollen cultured at 10°C or 35°C was significantly lower than that of pollen cultured at 25°C, indicating that both low temperature and high temperature adversely affect pollen germination. Therefore, the effect of temperature on pollen germination should be considered during cross-pollination of *E. racemosa*.

The pH value of the germination medium is the main factor that affects pollen germination in many plants (Munzuroglu et al., 2003; Burke et al., 2004). Zaman (2009) and Fragallah et al. (2019) reported that pH levels affected pollen germination *in vitro*. Fragallah et al. (2019) reported that pollen growth was optimal at pH 7.0 and worst at pH 5.3. A pH level of 8.5–9.0 was optimal for *in vitro* pollen germination in most cucurbit species (Zaman, 2009). In the present study, the pH value of the germination medium had a significant effect on the germination of *E. racemosa* pollen. This is the first report of the effects of different pH values on the germination of *E. racemosa*. The pollen germination rates increased and then decreased when the pH level increased from 4.5 to 8.0. The pollen germination rate was lowest when the pH of the medium was 4.5. The optimal pH value for pollen germination and pollen tube elongation was 6.5.

### Pollen storage

Pollen preservation is important for producing new varieties through hybridization (Báez et al., 2002; Kadri et al., 2022). Pollen longevity can be extended when it has a low moisture content and when stored at low temperatures (Akond et al., 2012; Özcan, 2020). Our results showed that *E. racemosa* pollen stored at four temperatures had lost some germination capacity compared to the fresh pollen. These results are consistent with several other studies in which pollen stored at 25–30°C or 3–4°C in a refrigerator showed lower germination rate than fresh pollen from *Prunus persica* (Devi et al., 2018), *Carya illinoinensis* (Wang et al., 2021) and *Phoenix dactylifera* (Shaheen, 1986; Mortazavi et al., 2010; Maryam et al., 2015). Our results showed that dried pollen still germinates at 50% after 390 days of storage at −80°C, which is the optimal temperature for storage of *E. racemosa.* This suggests that the longevity of *E. racemosa* pollen may exceed 12 months at −80°C. Storing pollen at −20°C would also be a simple way to help maintain pollen potency. *In vitro* germination was significantly higher for pollen stored at −20°C for 390 days compared to 4°C storage, while pollen stored at 25°C for 180 days lost its ability to germinate. These observations were similar to pollen germination from six male varieties of *P. dactylifera*, with a significantly faster reduction in *in vitro* germination at 25°C compared to pollen stored at 4°C or −20°C (Mesnoua et al., 2018b). Jia et al. (2020) and Liu et al. (2020) also reported that pollen viability was significantly higher after −20°C storage than 4°C storage. Our results are also consistent with those of Liu et al. (2020) and Jia et al. (2020), where a higher pollen *in vitro* germination rate was obtained at −20°C, followed by lower germination rates following storage at 4°C and 25°C (Maryam et al., 2015; Jaskani and Naqvi, 2017).

The method used to thaw frozen pollen has an important influence on its germination rate and depends on the plant species and the water content of the pollen (Sun et al., 1998; Xu et al., 2000; Xue et al., 2000). The thawing method is as important as the storage temperature (Wen et al., 1999). Wen et al. (1999) studied the vitrified callus of *Freesia refracta* and concluded that thawing should be rapidly conducted at 40°C. Based on a review of research on the cryopreservation of plant material, Kulus and Zalewska (2014) concluded that slow thawing is usually less effective and more time-consuming than rapid warming. The results of four thawing tests on *E. racemosa* showed that the pollen germination rate and pollen tube length when pollen was thawed at 35°C were significantly better than pollen thawed at 25 °C. These results are not consistent with the results reported by Xu et al. (2000); Xue et al. (2000) and Wang et al. (2021), indicating pollen grains of different species may have different responses to thawing temperatures.

## Conclusions

Red ink and TTC can effectively distinguish viable pollen from unviable pollen for *E. racemosa*. Sucrose in germination media can affect pollen germination. The best liquid medium for pollen germination was a medium containing 150 g· L^−1^ sucrose, 100 mg· L^−1^ boric acid, 150 mg· L^−1^ Ca(NO_3_)_2_ and 50 mg· L^−1^ GA_3_. This medium resulted in a germination rate of 80.18%. A temperature of 25°C was optimal for pollen germination and pollen tube growth for *E. racemosa*. The length of pollen tubes was four times longer at 25°C compared to lengths at 10°C. The optimal pH for *in vitro* pollen germination was 6.5. The longevity of dried pollen was longer than that of fresh pollen. Storage of *E. racemosa* dried pollen at −80°C preserved pollen germination more effectively than storage at 25°C, 4°C and −20°C. Pollen longevity at −80°C exceeded 365 days. Thawing in a 35°C water bath for 4 min was the best thawing method for pollen stored at −20°C and −80°C.This paper systematically evaluates the influences of the parameters on pollen viability, pollen germination and pollen longevity, and provides theoretical direction for cross-breeding research in *E. racemosa*.

## Data availability statement

The original contributions presented in the study are included in the article/supplementary material. Further inquiries can be directed to the corresponding authors.

## Author contributions

We conceived and designed the experiments; WJ, YW and DK performed the experiments. YW, ZM, ZW and DK analyzed the data. WJ, ZW, ZM and SH wrote and revised the manuscript. All authors contributed to the article and approved the submitted version.

## Funding

This work was supported by The National Key Research and Development Program of China (Grant no. 2018YFD1000401) and Henan Key Research and Development Plan (Grant no. 202102110082).

## Conflict of interest

The authors declare that the research was conducted in the absence of any commercial or financial relationships that could be construed as a potential conflict of interest.

## Publisher’s note

All claims expressed in this article are solely those of the authors and do not necessarily represent those of their affiliated organizations, or those of the publisher, the editors and the reviewers. Any product that may be evaluated in this article, or claim that may be made by its manufacturer, is not guaranteed or endorsed by the publisher.
